# An evaluation of the NOVA I ion selective electrode analyser for sodium and potassium determination

**DOI:** 10.1155/S1463924680000624

**Published:** 1980

**Authors:** W. Annan, N. A. Kirwan, W. S. Robertson, P. R. Teasdale, B. P. Ager

**Affiliations:** 1 Biochemistry Department Hull Royal Infirmary Hull HU3 2JZ UK; 2 Scientific and Technical Branch Department of Health and Social Security 14 Russell Square London UK


### An evaluation of the NOVA 1 ion selective electrode analyser for sodium and potassium determination

**W. Annan, N. A. Kirwan, W. S. Robertson, and P. R. Teasdale**
*Biochemistry Department, Hull Royal Infirmary, Hull HU3 2JZ*

**and B. P. Ager**
*Scientific and Technical Branch, Department of Health and Social Security, 14 Russell Square, London*

### Introduction

In the NOVA 1 sodium and potassium analyser*, ion-selective electrodes measure sodium and potassium by direct potentiometry in separated plasma or serum or in the plasma phase of whole blood and in urine after dilution. Sodium is measured using a glass electrode and potassium using a valinomycin electrode. The sodium, potassium and reference electrodes all have a straight tube design and these are connected to a sampling probe, peristaltic pump and reagent pack via tubing and a pinch valve system. Samples can be aspirated from sample cups, capillary tubes or syringes. The sample volume required is 300 $\mu l$ for blood, plasma or serum, or 100 $\mu l$ for urine.

The NOVA 1 is controlled by a microprocessor which initiates a two point calibration of the electrodes at 2 hourly intervals. A single point calibration with an aqueous standard is automatically carried out with the analysis of each specimen and the test result is displayed 58 seconds after the start of the sample aspiration cycle. The microprocessor also monitors the performance of the system and if a system fault is detected this is identified and a fault status code displayed.

Urine diluent, standards, reference solution and a waste container, are all supplied in a disposable reagent pack within the instrument. The main operating controls consist of two push buttons labelled 'calibrate' and 'analyze', and auxiliary controls are also present. These comprise of a series of push buttons for other functions such as purging the system using a fast pump speed, testing the system display, displaying output from electrodes in millivolts and setting up the instrument for analysis in either 'blood' or 'urine' mode. The measurement of sodium and potassium in blood, serum or plasma is made on an undiluted sample, whereas urine samples are automatically diluted before analysis with magnesium acetate solution. After the recommended daily calibration of the instrument, a sample can be analysed with the push of a single control button and results are displayed in approximately one minute.

### Materials

### Reagents

All reagents required for sample analysis and calibration were supplied in a fluids pack by NOVA Biomedical. These consisted of a magnesium acetate diluent (52 mmol/l), potassium chloride reference solution (2 mmol/1), standard solution A (sodium chloride 140 mmol/l, potassium chloride 4 mmol/l, magnesium acetate 3 mmol/l) and standard solution B (sodium chloride 50 mmol/l, potassium chloride 40 mmol/l, magnesium acetate 22 mmol/1). In addition NOVA Biomedical supplied a sodium electrode conditioning fluid (ammonium bifluoride 100 mmol/l) and four external standard solutions, with the following compositions:

1. Sodium chloride 150 mmol/l, potassium chloride 6 mmol/l;

2. Sodium chloride 120 mmol/l, potassium chloride 2 mmol/l, magnesium acetate 11 mmol/l;

3. Sodium chloride 100 mmol/l, potassium chloride 100 mmol/l;

4. Sodium chloride 10 mmol/l, potassium chloride 10 mmol/l.

### Quality control materials

The following quality control materials were used during the evaluation and were selected to provide low, medium and high concentrations of sodium and potassium;

Versatol, Versatol A and Versatol A Alternate (assayed human control sera) and Quality Assurance Serum II (unassayed human control serum) supplied by General Diagnostics, W. R. Warner & Co. Ltd.; Wellcomtrol 1 and Wellcome Autoset M (assayed bovine control sera) and Wellcomtrol 2 (assayed horse control serum) supplied by Wellcome Reagents Ltd.; Equitrol (unassayed horse control serum) supplied by Tissue Culture Services Ltd.; Preciflo calibration serum (assayed human control serum) supplied by Boehringer Corporation Ltd.; Hyland Q-Pak Automated (assayed human control serum) and Hyland Q-Pak (unassayed human urine control) supplied by Travenol Laboratories Ltd.; Fisher I and Fisher II (unassayed human urine controls) supplied by Fisher Scientific Company Diagnostics Division.

Each of the lyophilised quality control materials was reconstituted according to the manufacturer's instructions. Materials used for between batch precision assessment were dispensed into small plastic tubes and stored at -15°C.

### Methods

### Precision

Within-batch precision was assessed by replicate measurements of a selection of quality control materials (sera and urines) covering a range of concentrations of sodium and potassium and also heparinised whole blood obtained from hospital patients. Up to 20 measurements were made on each specimen within as short a time as possible.

Between-batch precision was assessed by replicate measurements of quality control materials (sera and urines) over a 20 day period. Each day an aliquot of each material was thawed, mixed and a single measurement made.

### Linearity

The linearity of the instrument for sodium and potassium, in both the blood and urine modes, was assessed by measurement of a range of aqueous standards and diluted

**Manufactured by NOVA Biomedical, Newton, Mass, USA, and marketed in the United Kingdom by American Hospital Supply (UK) Ltd., Didcot, Oxon.*

quality control materials. This assessment was carried out at the beginning, middle and end of the six week evaluation period.

**Correlation studies**

Plasma sodium and potassium were measured on 26 specimens from apparently healthy laboratory staff, 159 specimens from hospital patients and 20 lipaemic specimens from hospital patients, by the NOVA 1 and by an IL 343 flame photometer on a Vickers M300 Multichannel Analyser.

Urine sodium and potassium were measured on 100 specimens from hospital patients by the NOVA 1 and by a Technicon SMA 6/60 flame photometer. Materials from the Wellcome Group Quality Control Programme and the UK National Quality Control Scheme were analysed by the NOVA 1 and the results compared with the flame photometer group mean values.

Each of eight different commercial assayed quality control materials was analysed in duplicate by both the NOVA 1 and the IL 343 flame photometer. Results were compared with the manufacturers' stated values for sodium and potassium.

A comparison was made between the results from whole blood measured on the NOVA 1 and those from separated plasma from the same specimens also measured on the NOVA 1. The comparison was made on a group of 15 specimens which had been received for blood gas analysis, and also on a group of 21 specimens, collected into lithium heparin tubes, from apparently healthy laboratory staff.

**Table 1. Precision of NOVA 1 in blood mode, using quality control sera (n = 20 for each material)**

| | Sodium | | | Potassium | | |
|---|---|---|---|---|---|---|
| | Mean (mmol/l) | SD (mmol/l) | CV (%) | Mean (mmol/l) | SD (mmol/l) | CV (%) |
| Within-batch | | | | | | |
| Versatol A Alternate | 154.3 | 0.8 | 0.5 | 3.13 | 0.02 | 0.6 |
| Quality Assurance Serum II | 155.4 | 0.4 | 0.3 | 7.20 | 0.05 | 0.7 |
| Equitrol | 142.5 | 0.3 | 0.2 | 4.26 | 0.03 | 0.6 |
| Equitrol (diluted) | 118.9 | 0.5 | 0.4 | 3.56 | 0.03 | 0.8 |
| Between-batch | | | | | | |
| Versatol A Alternate | 154.0 | 0.7 | 0.5 | 3.09 | 0.03 | 1.0 |
| Quality Assurance Serum II | 154.7 | 0.7 | 0.5 | 7.21 | 0.08 | 1.2 |
| Equitrol | 141.7 | 0.8 | 0.6 | 4.18 | 0.06 | 1.4 |
| Equitrol (diluted) | 118.6 | 0.4 | 0.3 | 3.53 | 0.07 | 1.9 |

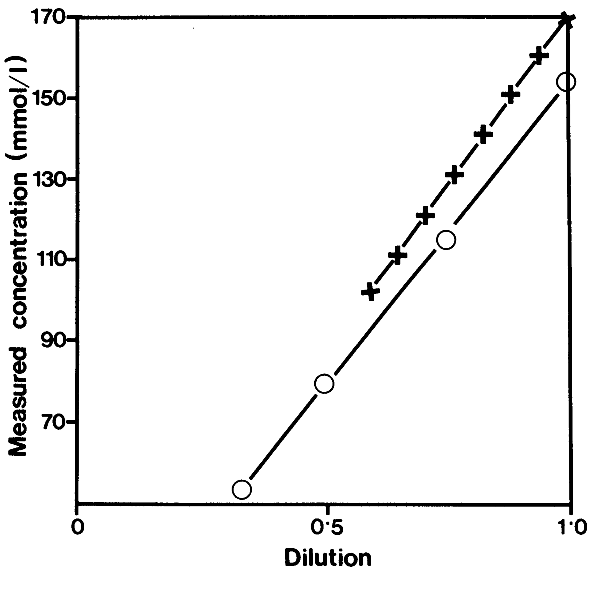

*Figure 1. Linearity of NOVA 1 sodium determination in the blood mode. (X = aqueous standards, O = quality control serum)*

**Statistical methods**

Calculation of the means, standard deviations, coefficients of variation, correlation coefficients and linear regression analysis were carried out by standard methods using a PDP 11/34 computer.

Comparison of the means was made using either paired or unpaired Student's 't' test, as appropriate.

### Results and discussion

**Precision**

The within-batch and between-batch precision figures for control sera for the NOVA 1 operating in the blood mode, are shown in Table 1. The average coefficients of variation for sodium were 0.4% (range 0.2 – 0.5) within-batch and 0.5% (range 0.3 – 0.6) between-batch. For potassium the average coefficients of variation were 0.7% (range 0.6 – 0.8) within-batch and 1.4% (range 1.0 – 1.9) between-batch. These were considered to be satisfactory.

The within-batch precision data of the NOVA 1 for whole blood is shown in Table 2. The average within-batch coefficient of variation for sodium was 0.3% (range 0.3 – 0.5) and for potassium 0.8% (range 0.4 – 1.4). These levels of precision were also considered to be satisfactory.

The between-batch precision for whole blood could not be determined because of the unstable nature of the sample material.

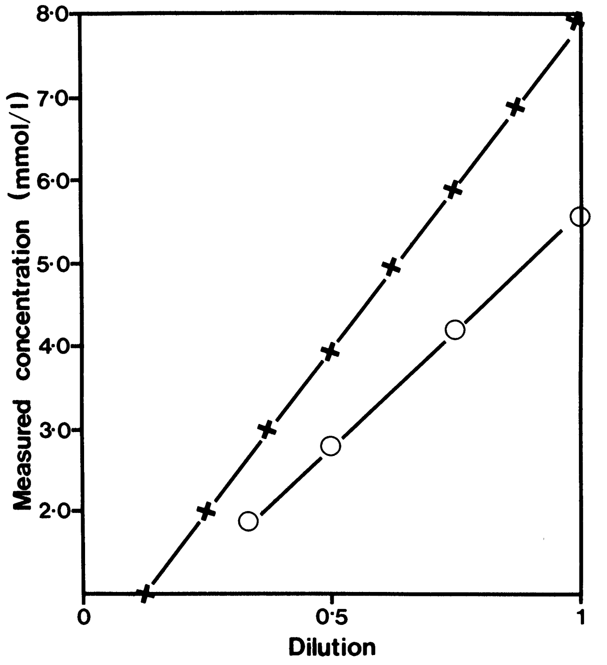

*Figure 2. Linearity of NOVA 1 potassium determination in the blood mode. (X = aqueous standards, O = quality control serum)*

The within-batch and between-batch precision figures for the NOVA 1 in the urine mode using urine quality control materials are shown in Table 3. The average coefficients of variation for sodium were 1.2% (range 0.7 – 1.6) within-batch and 2.1% (range 1.4 – 2.7) between-batch. The average within-batch coefficient of variation for potassium was 1.0% (range 1.0 – 1.1) and the average between-batch coefficient of variation was 2.7% (range 2.0 – 3.3). The authors consider the precision of the NOVA 1 for sodium and potassium in the urine mode to be satisfactory. The greater coefficients of variation of the instrument in the urine mode as compared to the blood mode may be due to the additional urine dilution step.

**Linearity**

Figures 1 to 4 show the results of linearity experiments. In each figure a point represents the mean of three measurements made at the beginning, middle and end of the evaluation period.

**Table 2. Within-batch precision of NOVA 1 in blood mode, using whole blood** (The results shown are for n replicate analyses of each of 10 different whole blood specimens)

| Sodium | | | Potassium | | | |
|---|---|---|---|---|---|---|
| Mean (mmol/l) | SD (mmol/l) | CV (%) | Mean (mmol/l) | SD (mmol/l) | CV (%) | n |
| 136.6 | 0.5 | 0.3 | 4.52 | 0.03 | 0.6 | 11 |
| 143.1 | 0.4 | 0.3 | 4.19 | 0.04 | 0.9 | 11 |
| 158.8 | 0.5 | 0.3 | 4.26 | 0.03 | 0.7 | 14 |
| 144.5 | 0.4 | 0.3 | 2.94 | 0.02 | 0.5 | 9 |
| 146.3 | 0.5 | 0.3 | 2.89 | 0.04 | 1.4 | 8 |
| 137.0 | 0.4 | 0.3 | 3.52 | 0.02 | 0.4 | 8 |
| 128.9 | 0.3 | 0.3 | 3.15 | 0.02 | 0.8 | 9 |
| 148.7 | 0.8 | 0.5 | 3.67 | 0.02 | 0.6 | 13 |
| 141.3 | 0.4 | 0.3 | 4.32 | 0.04 | 1.0 | 16 |
| 130.9 | 0.4 | 0.3 | 2.85 | 0.04 | 1.3 | 7 |

In the blood mode the NOVA 1 gave linear results for sodium (Figure 1) and potassium (Figure 2) over the ranges investigated (50 – 170 mmol/l and 1 – 8 mmol/l respectively). In the urine mode, linear results for sodium were obtained over the ranges 20 – 300 mmol/l for aqueous standards and 50 – 150 mmol/l for urine QC material (Figure 3). For potassium linearity was demonstrated over the ranges 20 – 150 mmol/l for aqueous standards and 20 – 70 mmol/l for urine QC material (Figure 4).

**Correlation Studies**

*a) Patient and normal plasma samples*

Table 4 shows the comparison of results obtained by the NOVA 1 and flame photometry on 26 plasma samples from apparently healthy laboratory staff (Group 1), 159 plasma samples from hospital patients (Group 2) and 20 lipaemic plasma samples from hospital patients (Group 3). In each group the sodium and potassium results by NOVA 1 are significantly higher than the corresponding results obtained by flame photometry. In Group 1 and Group 2 the means

**Table 3. Precision of NOVA 1 in urine mode, using urine quality control specimens** (n = 20 for each material)

| | Sodium | | | Potassium | | |
|---|---|---|---|---|---|---|
| | Mean (mmol/l) | SD (mmol/l) | CV (%) | Mean (mmol/l) | SD (mmol/l) | CV (%) |
| Within-batch | | | | | | |
| Fisher I | 156.5 | 2.5 | 1.6 | 70.2 | 0.8 | 1.1 |
| Hyland Q-Pak | 100.2 | 0.7 | 0.7 | 38.9 | 0.4 | 1.0 |
| Fisher II | 119.9 | 1.7 | 1.4 | 49.9 | 0.5 | 1.0 |
| Between-batch | | | | | | |
| Hyland Q-Pak | 99.7 | 2.7 | 2.7 | 38.7 | 1.3 | 3.3 |
| Fisher II | 117.6 | 1.7 | 1.4 | 49.6 | 1.0 | 2.0 |

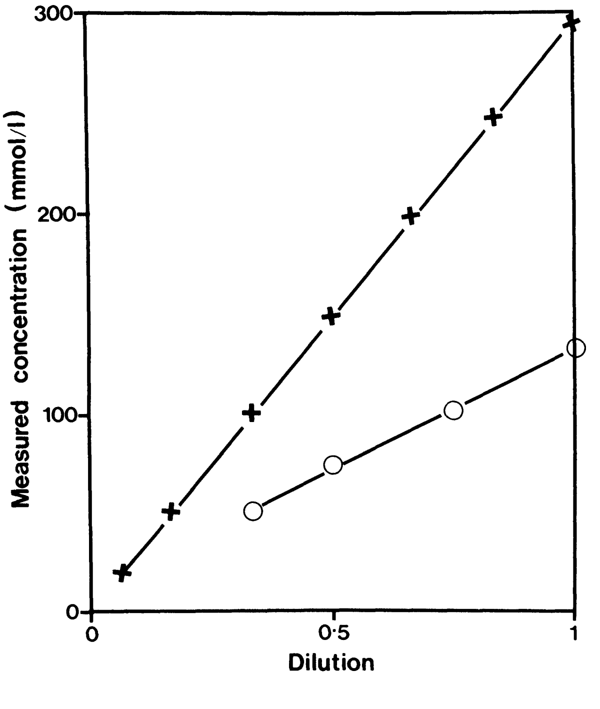

*Figure 3. Linearity of NOVA 1 sodium determination in the urine mode. (X = aqueous standards, O = quality control urine)*

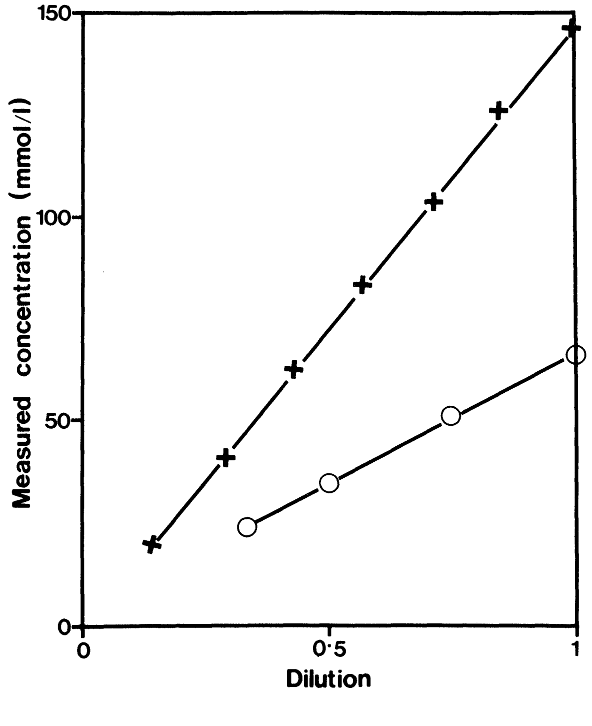

*Figure 4. Linearity of NOVA 1 potassium determination in the urine mode. (X = aqueous standards, O = quality control urine)*

of the differences between results by NOVA 1 and results by flame photometry were 3.7 mmol/l for sodium ($p<0.01$) and 0.05 mmol/l for potassium ($p<0.01$). Similar differences have been reported by Annan *et al* [1] and Ladenson [2]. In Group 3 the means of the differences were 4.3 mmol/l for sodium ($p<0.01$) and 0.10 mmol/l for potassium ($p<0.01$). The differences found in Group 3 are not significantly different from those found in Groups 1 and 2. Figures 5 and 6 show the correlation and linear regression analysis of the patient data in Group 2 for sodium and potassium respectively.

*b) Patient urine samples*

The comparison of results obtained by flame photometry (SMA 6/60) and the NOVA 1 on 100 urines from hospital patients is shown in Table 5. Figures 7 and 8 show the correlation and linear regression analysis for sodium and potassium respectively. For sodium there is no significant difference between results obtained by the NOVA 1 and those by flame photometry.

Analysis of the potassium data shows that the NOVA 1 values are significantly lower than the flame photometer results (mean of differences = 1.5 mmol/l, $p<0.01$). All the urine potassium measurements, which covered a range of concentrations from 2.7 – 79.0 mmol/l, were made on samples which were not diluted before being presented to the NOVA 1. The NOVA 1 Instruction Manual states that occasionally urines with potassium values greater than 50 mmol/l give results 5% to 10% lower than flame photometry and that this is attributable to a weak binding agent for potassium in these samples. Consequently the Instruction Manual recommends that urines with potassium values greater than 45 mmol/l should be diluted 1 in 5 with distilled water before analysis. The data shown in Figure 8 supports this recommendation.

*c) Quality control schemes*

Table 6 shows results for sodium and potassium obtained by the NOVA 1 on materials from the Wellcome and UK National Quality Control Schemes. The IL flame photometer

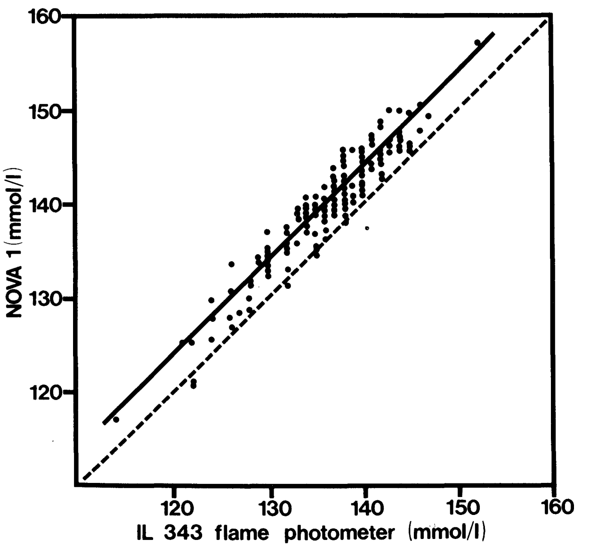

*Figure 5. Comparison of sodium results by flame photometer (IL 343) and NOVA 1 on 159 plasma specimens from hospital patients. ($r = 0.957$, $y = 0.998x + 4.0$)*

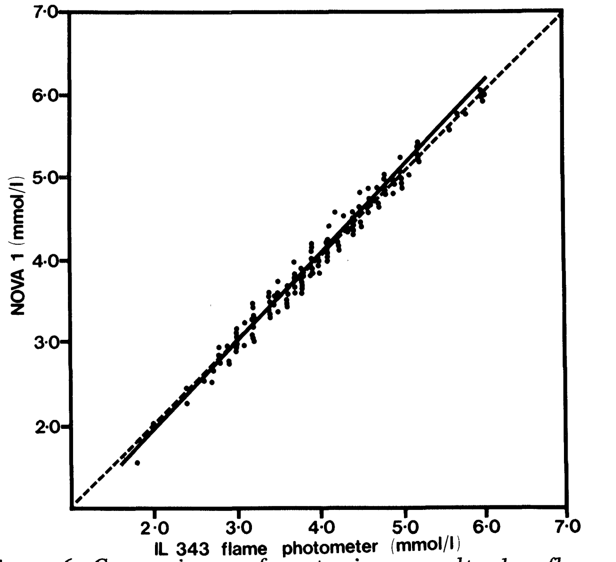

*Figure 6. Comparison of potassium results by flame photometer (IL 343) and NOVA 1 on 159 plasma specimens from hospital patients. ($r = 0.900$, $y = 1.029x - 0.06$)*

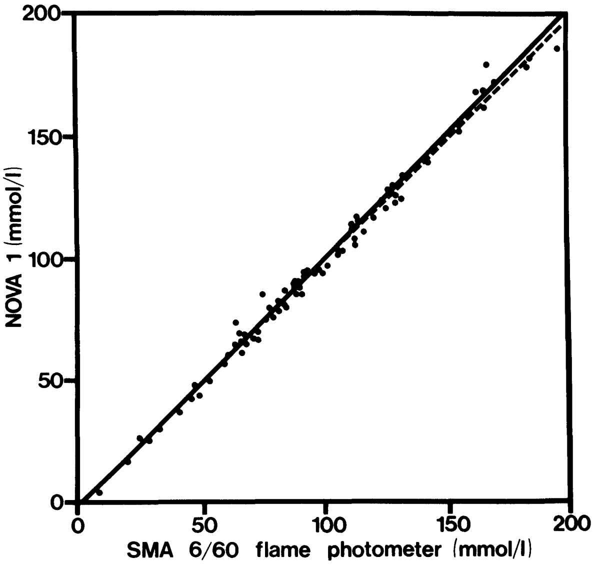

*Figure 7. Comparison of sodium results by flame photometer (SMA 6/60) and NOVA 1 and 100 urine specimens from hospital patients. ($r = 0.996$, $y = 1.012x - 0.7$)*

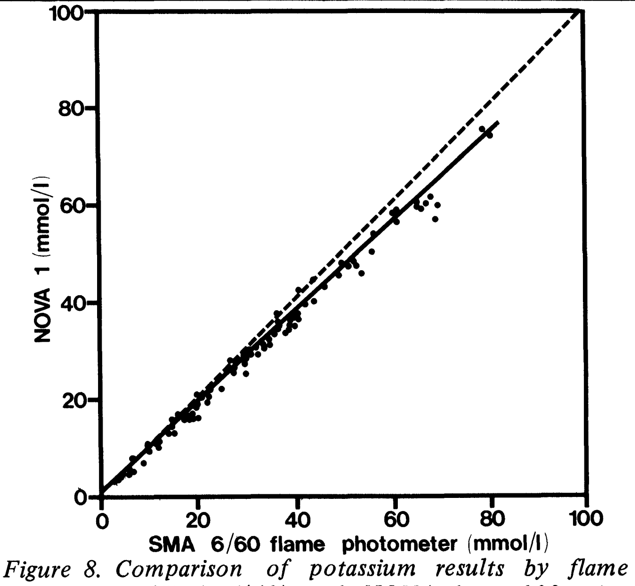

*Figure 8. Comparison of potassium results by flame photometer (SMA 6/60) and NOVA 1 on 100 urine specimens from hospital patients. ($r = 0.992$, $y = 0.914x + 1.35$)*

group mean results from the relevant quality control scheme are also shown.

In general the NOVA 1 results for potassium show good agreement with the QC group mean values. Sodium results on the NOVA 1 results show differences from the flame photometer group mean values which range from -0.1 mmol/l to +4.1 mmol/l.

*d) Commercial quality control sera*

The results shown in Table 7 were obtained from the analysis of eight commercial quality control materials for sodium and potassium by NOVA 1 and flame photometry (IL 343). These results are compared with the manufacturers' stated values. Each NOVA 1 and flame photometer result is the mean of duplicate determinations.

All the control materials were freeze dried preparations which were reconstituted before use according to the manufacturers' instructions. Versatol, Versatol A, Versatol A Alternate, Wellcomtrol 1 and Wellcomtrol 2 were reconstituted with distilled water. The manufacturer's diluent for reconstituting Wellcome Autoset M was an aqueous solution of sodium bicarbonate; that for Hyland Q-Pak Automated was a buffer solution containing trimethyl ammonium bicarbonate and glycerol; and that for Preciflo was an aqueous solution containing caesium chloride.

The results in Table 7 show that flame photometry produced sodium and potassium values for the eight QC materials which were in close agreement with the manufacturers' stated values, but that the NOVA 1 produced anomalous results for some materials. For Preciflo, the NOVA 1 gave a sodium result 6.4 mmol/l lower, and a potassium result 11.29 mmol/l higher, than the stated values. For Hyland Q-Pak Automated, a sodium result 3.8 mmol/l lower than the manufacturer's value was obtained on the NOVA 1. These anomalies are probably due to interference with the performance of the electrodes by components in the reconstituting fluids of these materials.

*e) Whole blood and plasma*

Table 8 shows the results for sodium and potassium measured by the NOVA 1 on whole blood compared to the results obtained on separated plasma from the same specimens. The data shown is for 15 specimens from hospital patients (Group 4), and for 21 specimens from apparently healthy laboratory staff (Group 5). The specimens in Group 4 were received for blood gas analysis and contained variable amounts of sodium heparin as anticoagulant. The specimens in Group 5 were collected into sample tubes containing lithium heparin. In each group the values of sodium and potassium on plasma specimens were significantly higher than those obtained on whole blood. The mean differences between plasma and whole blood results in Group 4 were 1.3 mmol/l for sodium ($p<0.01$) and 0.04 mmol/l for potassium ($p<0.05$). In Group 5 the mean differences were 1.6 mmol/l for sodium ($p<0.01$) and 0.04 mmol/l for potassium ($p<0.01$).

The NOVA 1 Instruction Manual states that since the electrodes do not respond to sodium or potassium in the cellular components of blood, the instrument will give results for whole blood which are identical to those obtained on plasma from the same sample. There are small, but statistically significant differences, between results obtained on whole blood and those obtained on separated plasma.

**Table 4. Comparison of NOVA 1 and IL 343 flame photometer plasma sodium and potassium determinations**

Group 1: 26 specimens from apparently healthy laboratory staff.

Group 2: 159 specimens from hospital patients.

Group 3: 20 lipaemic specimens from hospital patients.

| | Sodium (mmol/l) | Potassium (mmol/l) |
|---|---|---|
| Group 1 (n = 26) | | |
| NOVA 1 mean (± SD) | 142.8 (± 1.8) | 4.07 (± 0.38) |
| IL 343 mean (± SD) | 139.1 (± 1.4) | 4.02 (± 0.33) |
| Mean of differrences (± SD) | 3.7 (± 1.3)** | 0.05 (± 0.09)** |
| Group 2 (n = 159) | | |
| NOVA 1 mean (± SD) | 139.8 (± 6.2) | 3.98 (± 0.83) |
| IL 343 mean (± SD) | 136.1 (± 6.0) | 3.93 (± 0.80) |
| Mean of differences (± SD) | 3.7 (± 1.8)** | 0.05 (± 0.12)** |
| Group 3 (n = 20) | | |
| NOVA 1 mean (± SD) | 143.8 (± 4.8) | 4.33 (± 0.76) |
| IL 343 mean (± SD) | 139.5 (± 4.8) | 4.23 (± 0.69) |
| Mean of differences (± SD) | 4.3 (± 1.7)** | 0.10 (±0.13)** |

** $p<0.01$

**Table 5. Comparison of NOVA 1 and SMA 6/60 flame photometer sodium and potassium determinations in 100 urine specimens from hospital patients**

| | Sodium (mmol/l) | Potassium (mmol/l) |
|---|---|---|
| NOVA 1 mean (± SD) | 101.2 (± 50.1) | 32.0 (± 16.0) |
| SMA 6/60 mean (± SD) | 100.7 (± 49.3) | 33.5 (± 17.3) |
| Mean of differences (± SD) | 0.5 (± 4.4)NS | 1.5 (± 2.5)** |

NS Not Significant

** $p<0.01$

**Table 6. Comparison of results by NOVA 1 with IL flame photometer group means in the Wellcome and UK National Quality Control Schemes, for sodium and potassium**

| QC Scheme | Sodium (mmol/l) | | Potassium (mmol/l) | |
|---|---|---|---|---|
| | NOVA 1 | Group Mean (SD) | NOVA 1 | Group Mean (SD) |
| Wellcome | 148.8 | 144.7 (2.3) | 4.51 | 4.43 (0.10) |
| National | 155.5 | 152.9 (2.0) | 5.75 | 5.70 (0.11) |
| Wellcome | 123.6 | 123.4 (1.9) | 3.48 | 3.49 (0.07) |
| Wellcome | 143.4 | 140.9 (2.1) | 5.19 | 5.10 (0.11) |
| National | 129.7 | 128.5 (1.6) | 3.47 | 3.47 (0.08) |
| Wellcome | 154.2 | 151.8 (2.1) | 5.41 | 5.54 (0.12) |
| National | 150.6 | 150.6 (2.3) | 7.10 | 7.10 (0.19) |

**Table 7. Comparison of manufacturers' quoted values for sodium and potassium in assayed quality control materials with the means of duplicate determinations for sodium and potassium by NOVA 1 and IL 343 flame photometer**

| Material | Sodium (mmol/l) | | | Potassium (mmol/l) | | |
|---|---|---|---|---|---|---|
| | Manufacturer's value | NOVA 1 | IL 343 | Manufacturer's value | NOVA 1 | IL 343 |
| Versatol | 142 | 143.6 | 141.0 | 5.0 | 4.95 | 4.95 |
| Versatol A | 129 | 129.0 | 128.5 | 7.3 | 7.05 | 7.25 |
| Versatol A Alternate | 153 | 152.6 | 152.0 | 3.1 | 3.12 | 3.15 |
| Wellcome 1 | 143 | 143.6 | 143.0 | 4.9 | 4.81 | 4.90 |
| Wellcome Autoset M | 147 | 148.7 | 147.5 | 5.7 | 5.72 | 5.65 |
| Preciflo | 138 | 131.6 | 140.0 | 5.1 | 16.39 | 5.40 |
| Hyland Q-Pak Automated | 148 | 144.2 | 150.0 | 5.1 | 4.85 | 5.00 |
| Wellcome 2 | 149 | 148.1 | 149.0 | 5.6 | 5.48 | 5.55 |

### Other investigations

*a) Probe position*

The NOVA 1 sampling probe has two possible operating modes, a 'down' position for aspiration of samples from specimen cups, and an 'up' position for aspiration of samples from syringes or capillary tubes. The two sampling positions were compared by analysis of two quality control sera twenty times each in each sampling position. The differences in the mean sodium and potassium values for each sampling position were compared and the results are shown in Table 9. The sodium values with the probe in the 'up' position were significantly higher than those in the 'down' position. Specimen 1 showed a difference between the means of 0.7 mmol/l ($p<0.01$) and specimen 2 a difference of 0.8 mmol/l ($p<0.01$).

For potassium, significantly higher results were also obtained with the probe in the 'up' position. For specimen 1 the difference between the the means ('up' – 'down') was 0.02 mmol/l ($p<0.05$) and for specimen 2 the difference was 0.03 mmol/l ($p<0.01$). The authors are unable to explain why these differences should exist.

All of the analyses carried out during the evaluation period were performed with the probe in the 'up' position with the exception of the correlation studies performed on Groups 1 and 2, described earlier.

*b) Heparin*

The NOVA 1 Instruction Manual recommends that blood samples be collected with sodium heparin (10–20 units/ml) as anticoagulant, noting that sodium results will be elevated by approximately 1 mmol/l. The majority of the measurements on heparinised plasma samples were carried out on specimens containing lithium heparin but some determinations were also made on samples containing other forms of heparin. An initial investigation on whole blood samples collected for blood gas analysis showed some anomalous results for NOVA 1 sodium and potassium compared to the flame photometer results. The authors were interested in the effect of heparin in blood samples collected anaerobically for blood gas analysis and whether such samples were suitable for determination of sodium and potassium by the NOVA 1. Specimens for blood gas analysis are usually collected into syringes in which the dead space (needle + syringe end) is filled with a solution of sodium heparin. Since the dead space, the concentration of sodium heparin and the volume of blood collected may all vary, any effects on the measurement of sodium and potassium could also vary.

**Table 8. Comparison of results obtained from whole blood with those from separated plasma from the same specimen, for sodium and potassium, using the NOVA 1**

Group 4: 15 specimens from hospital patients. These specimens were received for blood gas analysis and contained variable amounts of sodium heparin.

Group 5: 21 specimens from apparently healthy laboratory staff. These specimens were collected into standard 10 ml lithium heparin tubes.

| | Sodium (mmol/l) | Potassium (mmol/l) |
|---|---|---|
| Group 4 (n = 15) | | |
| Whole blood mean (± SD) | 139.9 (± 8.8) | 3.45 (± 0.50) |
| Plasma mean (± SD) | 141.2 (± 9.1) | 3.49 (± 0.54) |
| Mean of differences (±SD) | 1.3 (± 0.8)** | 0.04 (±0.07)* |
| Group 5 (n = 21) | | |
| Whole blood mean (± SD) | 142.6 (± 1.7) | 3.99 (± 0.24) |
| Plasma mean (± SD) | 144.2 (± 1.8) | 4.03 (± 0.26) |
| Mean of differences (± SD) | 1.6 (± 0.7)** | 0.04 (± 0.04)** |

* $p<0.05$
** $p<0.01$

The effects of sodium heparin (5,000 units /ml heparin, 150 mmol/l sodium; and 25,000 units/ml heparin, 750 mmol/l sodium) and calcium heparin (25,000 units/ml) were investigated by adding 2 ml aliquots of a freshly collected whole blood specimen to a series of tubes containing varying amounts of these anticoagulants (from 20 $\mu$l to 200 $\mu$l). Table 10 shows results obtained on plasma samples containing varying amounts and types of heparin.

The main effects are an increase in heparin concentration which produces a decrease in sodium and potassium values measured by NOVA 1, and increasing sodium concentration in the form of 25,000 units/ml sodium heparin producing an increase in the sodium values measured by NOVA 1 and flame photometry, although the increase in the NOVA 1 values are less than those for flame photometry as a result of the heparin supression effect.

The type and volume of heparin and the volume of blood collected should be carefully controlled, otherwise specimens of blood collected for blood gas analysis may not be suitable for the determination of sodium and potassium by the NOVA 1.

### General comments

### Instrument use

In general, the NOVA 1 is simple to operate and has some particularly useful features. The results determined on a specimen are displayed constantly until the next sample is aspirated, so that if necessary an operator can aspirate a specimen and leave the instrument. The system of status codes is useful for identifying possible faults and in addition, results which may be unreliable (for example, due to the presence of air in the sample) are presented as a flashing display.

### Instruction manual

The NOVA 1 Instruction Manual is comprehensive and easy to follow. Included in the manual is a section on problem solving which utilises a series of flow diagrams as an aid to the diagnosis and correction of each fault identified by a status code. This was found to be particularly useful.

### Daily calibration

The Instruction Manual contains a section describing a series of checks and a calibration procedure to be followed each day. These checks were performed throughout the evaluation period and a record was maintained of the values recorded each day on four external standard solutions. These standards (two analysed in the blood mode and two in the urine mode) have limits ascribed to their values, within which results are considered to be acceptable.

At the beginning of the evaluation period results on the external standards were well within these limits, but as the

**Table 9. NOVA 1 sodium and potassium results on two quality control sera**

Comparison of "up" and "down" positions of sampling probe. For each specimen, n = 20 for each probe position.

| | Sodium (mmol/l) | Potassium (mmol/l) |
|---|---|---|
| Specimen 1 | | |
| Mean (± SD) 'up' | 142.3 (± 0.3) | 4.27 (± 0.03) |
| Mean (± SD) 'down' | 141.6 (± 0.4) | 4.25 (± 0.03) |
| Difference ('up'–'down') | 0.7** | 0.02* |
| Specimen 2 | | |
| Mean (± SD) 'up' | 119.0 (± 0.5) | 3.56 (± 0.03) |
| Mean (± SD) 'down' | 118.2 (± 0.5) | 3.53 (± 0.02) |
| Difference ('up'–'down') | 0.8** | 0.03** |

* $p<0.05$
**$p<0.01$

level of reagents in the fluid pack approached that at which replacement was recommended, potassium results on the standards analysed in the blood mode fell below the acceptable limits. Replacement of the fluids pack overcame this problem.

### Instrument faults

The fault status code most commonly displayed referred to instability of sodium reading, for which the recommended action is to treat the electrode with sodium electrode conditioning fluid. In almost every instance a repeat determination following this treatment gave a satisfactory result. This fault occurred only rarely with urine, plasma or serum specimens, but was common when specimens of whole blood were analysed.

During the evaluation the NOVA 1 was out of action for a period of four consecutive days, when a fault occurred which could not be corrected without recourse to American Hospital Supply Ltd., the United Kingdom distributors of NOVA 1. In this instance an electronic fault necessitated replacement of a circuit board and this could not be identified by following the problem solving guide in the Instruction Manual.

### Interfering substances

Ladenson [2] has reported that increasing the sodium concentration from 119 to 171 mmol/l had no effect on the potassium values, and that increasing the potassium concentration from 3.5 to 13.1 mmol/l had no effect on the sodium values. He also reported that no interference was found from creatinine, uric acid, urea, calcium, lithium and magnesium, nor from the range of pH values found in plasma.

Some anomalous results were produced by the NOVA 1 for sodium and potassium determinations in some commercial quality control materials and it is thought that certain constituents in the diluents of these materials, for example, trimethyl ammonium bicarbonate, glycerol or caesium chloride, interfere with the performance of the electrodes. In addition, the Instruction Manual states that the use of standard solutions containing viscosity adjusters and wetting agents could interfere with the NOVA 1 electrode performance and that the use of external standards other than those manufactured by NOVA Biomedical could void the electrode warranty. The investigation into the effects of heparin indicates that heparin can also have an effect on the electrode performance in the NOVA 1, particularly if it is present in high concentrations.

For the analysis of whole blood, for almost 50% of the specimens analysed, the NOVA 1 produced an error code indicating 'instability of sodium reading'. Repeat analysis of these specimens generally produced satisfactory results. This problem was not seen when separated plasma or serum samples were being analysed, nor when urine samples were being analysed in the urine mode. It was also noted that for some grossly lipaemic samples, no sodium or potassium results could be obtained on the NOVA 1 as the instrument continually produced an error code which represented 'no air when required' in the flow path of the instrument.

### Conclusions

The NOVA 1 sodium and potassium analyser is simple and easy to operate. It has the advantage over flame photometry that gas and a compressed air supply are not required. When the instrument is left in the 'Stat' mode it is immediately available for analytical determinations at the touch of a single 'Analyze' button.

**Table 10. Effect of heparin on sodium and potassium results determined in plasma from aliquots of a freshly collected blood sample, by NOVA 1 and IL 543 flame photometer**

a. 10 µl heparin per ml blood (equivalent to approximately 10 ml blood in heparinised syringe)
b. 20 µl heparin per ml blood (equivalent to approximately 5ml blood in heparinised syringe)
c. 40 µl heparin per ml blood (equivalent to approximately 2.5 ml blood in heparinised syringe)
d. 100 µl heparin per ml blood (equivalent to approximately 1 ml blood in heparinised syringe)

| | Sodium (mmol/l) | | Potassium (mmol/l) | |
|---|---|---|---|---|
| Sodium heparin (5,000 units/ml 150 mmol/l sodium) | NOVA 1 | IL 543 | NOVA 1 | IL 543 |
| a | 144.7 | 140.1 | 3.84 | 3.74 |
| b | 144.8 | 139.9 | 3.83 | 3.75 |
| c | 144.0 | 140.9 | 3.71 | 3.73 |
| d | 139.7 | 144.3 | 3.56 | 3.85 |
| Sodium heparin (25,000 units/ml 750 mmol/l sodium) | | | | |
| a | 149.3 | 148.2 | 3.64 | 3.72 |
| b | 152.2 | 157.2 | 3.37 | 3.64 |
| c | 157.8 | 176.1 | 3.03 | 3.58 |
| d | 174.0 | 223.1 | 2.45 | 3.49 |
| Calcium heparin (25,000 units/ml) | | | | |
| a | 140.6 | 137.0 | 3.77 | 3.72 |
| b | 137.2 | 135.0 | 3.72 | 3.78 |
| c | 132.2 | 132.6 | 3.53 | 3.73 |
| d | 118.0 | 122.7 | 3.05 | 3.44 |

The precision of the instrument both within- and between-batch is satisfactory. There is, however, a significant difference between NOVA 1 and flame photometer results for plasma sodium and potassium and the results confirm those reported by Ladenson [2]. The differences for potassium are small and possibly not clinically significant. The differences for sodium, however, are such that results obtained by NOVA 1 are not directly comparable to those obtained by flame photometry. These differences cannot be explained simply on the basis of the lipid and protein content of plasma and it is recommended that new reference ranges should be established for plasma sodium determined by NOVA 1.

The diluents of certain commercial quality control materials contain substances which interfere with the performance of the NOVA 1 electrodes. Heparin in high concentration can also affect the electrode performance and unless the heparin concentration in blood gas samples is carefully controlled then these samples may not be suitable for sodium and potassium determination by the NOVA 1.

ACKNOWLEDGEMENTS

The authors wish to acknowledge the financial support of the Scientific and Technical Branch of the Department of Health and Social Security who purchased the instrument and reagents used in the evaluation. Professor T. P. Whitehead and Dr M. A. Cresswell are thanked for permission to publish data from the National Quality Control Scheme and the Wellcome Quality Control Programme.

REFERENCES

[1] Annan, W., Kirwan, N. A., and Robertson, W. S., *Clinical Chemistry*, 1979, **25**, 643.
[2] Ladenson, J. H., *Clinical Chemistry*, 1979, **25**, 757.

